# EGR1 modulated LncRNA HNF1A-AS1 drives glioblastoma progression via miR-22-3p/ENO1 axis

**DOI:** 10.1038/s41420-021-00734-3

**Published:** 2021-11-12

**Authors:** Chunchun Ma, Hongliang Wang, Gang Zong, Jie He, Yuyang Wang, Fan Yang, Zhihao Yang, Erbao Bian, Bing Zhao

**Affiliations:** 1 Department of Neurosurgery, The Second Affiliated Hospital of Anhui Medical University, Hefei, 230601 China; 2 Cerebral Vascular Disease Research Center, Anhui Medical University, Hefei, 230601 China

**Keywords:** Long non-coding RNAs, Cell invasion, Cell growth

## Abstract

Accumulating evidences revealed that long noncoding RNAs (lncRNAs) have been participated in cancer malignant progression, including glioblastoma multiforme (GBM). Despite much studies have found the precise biological role in the regulatory mechanisms of GBM, however the molecular mechanisms, particularly upstream mechanisms still need further elucidated. RT-QPCR, cell transfection, western blotting and bioinformatic analysis were executed to detect the expression of EGR1, HNF1A-AS1, miR-22-3p and ENO1 in GBM. Cell proliferation assay, colony formation assay, wound healing, migration and invasion assays were performed to detect the malignant characters of GBM cells. The molecular regulation mechanism was confirmed by luciferase reporter assay, ChIP and RIP. Finally, orthotopic mouse models were established to examine the effect of HNF1A-AS1 in vivo. In the current study, we analyzed clinical samples to show that the HNF1A-AS1 expression is upregulated and associated with poor patient survival in GBM. Functional studies revealed that HNF1A-AS1 knockdown markedly inhibits malignant phenotypes of GBM cells, whereas overexpression of HNF1A-AS1 exerts opposite effect. Mechanistically, the transcription factor EGR1 forced the HNF1A-AS1 expression by directly binding the promoter region of HNF1A-AS1. Furthermore, combined bioinformatics analysis with our mechanistic work, using luciferase reporter assays and RIP, we first demonstrated that HNF1A-AS1 functions as a competing endogenous RNA (ceRNA) with miR-22-3p to regulate ENO1 expression in GBM cells. HNF1A-AS1 directly binds to miR-22-3p and significantly inhibits miR-22-3p expression, while ENO1 expression was increased. miR-22-3p inhibitor offsets the HNF1A-AS1 silencing induced suppression in malignant behaviors of GBM cells. ENO1 was verified as a direct target of miR-22-3p and its expression levels was negatively with the prognosis in GBM patients. Taken together, our study illuminated the definite mechanism of HNF1A-AS1 in promoting GBM malignancy, and provided a novel therapeutic target for further clinical application.

## Introduction

GBM, the most aggressive subtype of glioma in adults, highly malignant and high risk of recurrence, accounting for 47.1% of all malignant tumors of the nervous system [1]. In the light of the World Health Organization (WHO) classification of tumor in the central nervous system (CNS), GBM is classified as a grade IV glioma, with an unfavorable prognosis and a five-year overall survival rate less than 10% [2, 3]. Even after multimodal therapies, including maximal surgical resection, adjuvant radiotherapy, temozolomide (TMZ)-based chemotherapy or targeted therapy with rituximab, which have been commonly used in GBM patients, the patients’ overall survival rate is still unsatisfactory with a median survival time of 12–15 months from first diagnosis [4]. Therefore, it is extremely vital for us to establish new targeted therapies and to gain a clear understanding of the definite mechanisms of GBM malignant progression for the identification of new diagnostic and prognosis markers.

LncRNAs comprise a class of transcripts that are over 200 nt in length, which have many ways of regulating ability except for none protein-coding potential [5, 6]. The functions and mechanisms of lncRNAs exert their biological role through diverse modes, including chromatin modification, alternative splicing, mRNA stability, encode functional micropeptides, ceRNA molecular sponge to miRNAs, and so on [7–14]. An increasing number of reports have demonstrated that lncRNAs play a vital role in cancer progression, and are dysregulated in various human cancers, including GBM [15–17]. For example, LncRNA miR155HG is overexpressed in GBM, and promotes GBM progression by acting as a ceRNA for the tumor suppressor miR-185 to upregulate ANXA2 [18]. LncRNA AC016405.3 endpin cell proliferation and metastasis by regulating TET2, via sponging of miR-19a-5p in GBM cells [19]. LncRNA HOTAIRM1 is highly upregulated in GBM, which is positively correlated with tumor grade in patients with glioma, and aggravates the progression of GMB by regulating HOXA1 gene [20]. Despite several lncRNAs have been well studied, the functional mechanism of most lncRNAs in GBM remain largely unknow.

Hepatocyte nuclear factor 1 homeobox A antisense RNA 1 (HNF1A-AS1), was first identified as a lncRNA that upregulated in esophageal adenocarcinoma [21], and its overexpression was drastically associated with tumor advanced-stage and unfavorable outcomes in various cancers, including oral squamous carcinoma, urothelial carcinoma of the bladder and lung carcinoma, indicating an oncogenic function of HNF1A-AS1 in tumor progression [22–24]. However, the functional mechanism of HNF1A-AS1 in GBM has not been fully revealed yet. Herein, we demonstrate that HNF1A-AS1 is highly overexpressed in GBM tissues and cells, associates with poor patient survival, by acting as ceRNA for miR-22 and facilitating ENO1, to promote GBM malignant phenotypes. Therefore, these results imply that HNF1A-AS1 may serve as a druggable target for GBM.

## Results

### HNF1A-AS1 is upregulated in GBM and negatively related with patient prognosis

**Fig. 1:**
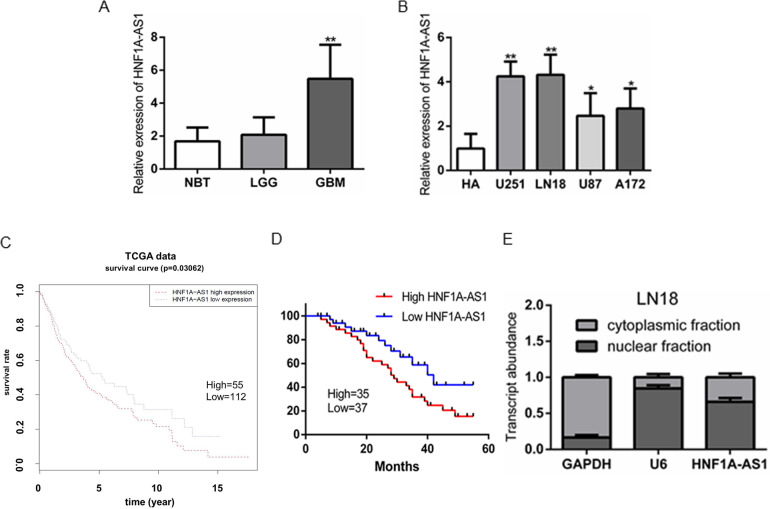
HNF1A-AS1 was highly expressed in GBM. **A** RT-QPCR analysis of HNF1A-AS1 expression in normal brain tissues (NBT) (*n* = 15), low-grade glioma tissues (*n* = 41) and GBM tissues (*n* = 72). **P < 0.01 vs. NBT group. **B** RT-QPCR analysis the expression levels of HNF1A-AS1 in normal human astrocyte (HA) and four GBM cell lines (U251, LN18, U87 and A172). *P < 0.05, **P < 0.01 vs. HA group. **C** In TCGA date, the overall survival rate of GBM patients with high (HNF1A-AS1-high, *n* = 55) and low (HNF1A-AS1-low, *n* = 112) expression of HNF1A-AS1 in tumor. **D** Kaplan–Meier analyses of the associations between HNF1A-AS1 expression level and overall survival of patients with human glioma in our department (The log-rank test was used to calculate *P*-values). **P < 0.01 vs. low HNF1A-AS1 expression group. **E** Nuclear and cytoplasmic fractions of HNF1A-AS1 in GBM cell lysates were analyzed by RT-QPCR.

To identify the expression of HNF1A-AS1 in GBM, using RT–QPCR analysis, we firstly measured HNF1A-AS1 expression in 15 normal brain tissues, 41 low-grade glioma tissues, 72 GBM tissues. We found that HNF1A-AS1 was obviously upregulated in GBM, as compared to low-grade glioma tissues and normal brain tissues, but there was no significant difference between low-grade glioma tissues and normal brain tissues (Fig. 1A). Furthermore, we also tested HNF1A-AS1 expression in normal human astrocyte (HA) and four GBM cell lines (U251, LN18, U87 and A172). The results indicated that HNF1A-AS1 was highly upregulated in four GBM cell lines in comparison with HA (Fig. 1B).

To further clarify the clinical significance of HNF1A-AS1 in GBM patients. The Kaplan–Meier method and log-rank test were performed to assess the expression of HNF1A-AS1 in GBM TCGA date cohort and our clinic study. TCGA date cohort demonstrated that GBM patients with high expression of HNF1A-AS1 was negatively associated with overall survival time (Fig. 1C). In our study, we also observed the similar results, indicating that high HNF1A-AS1 expression was significantly correlated with poor survival patients (Fig. 1D). In addition, subcellular fractionation and RT–QPCR analyses showed that HNF1A-AS1 is localized both in the cytoplasm and nucleus of GBM cells (Fig. 1E), which indicated its complicated functions.

### HNF1A-AS1 underpins GBM cells proliferation, migration, invasion

**Fig. 2:**
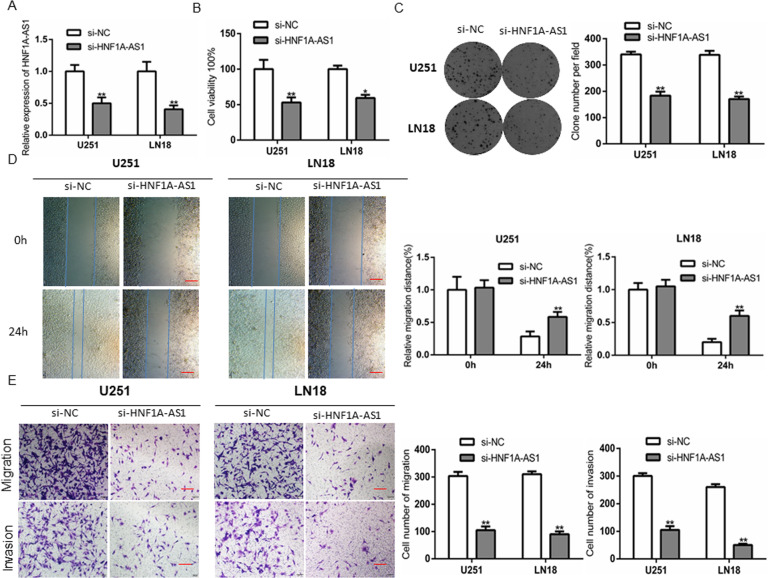
Knockdown of HNF1A-AS1 inhibited the proliferation, migration, and invasion of glioma cells in vitro. **A** Relative expression levels of HNF1A-AS1 after GBM cells transfected with si-HNF1A-AS1 and si-NC. **P < 0.01 vs. si-NC. **B** CCK-8 assay was performed to determine the proliferation effect of si-HNF1A-AS1 and si-NC transfected U251 and LN18 cells. *P < 0.05, **P < 0.01 vs. si-NC group. **C** Colony formation assay was performed to detect the proliferation of U251 and LN18 cells after transfected with si-HNF1A-AS1 and si-NC. **P < 0.01 vs. si-NC group. **D** Wound healing assay to evaluate the effect of HNF1A-AS1 on cell migration in U251 and LN18 cells (scale bar: 200 μm). **P < 0.01 < 0.05 vs. si-NC group. **E** Transwell assay for testing cell migration and invasion capacity (scale bar: 200 μm). **P < 0.01 vs. si-NC group. Data are presented as mean ± SD from three independent experiments.

To evaluate the biofunctional role of HNF1A-AS1 in GBM cells, the GBM cells were transfected with si-HNF1A-AS1 or si-NC. RT–QPCR was performed to examine the knockdown efficiency of HNF1A-AS1 after transfection 48 h (Fig. 2A). Compared with si-NC groups, CCK-8 assays revealed that HNF1A-AS1 knockdown significantly decreased cell proliferation (Fig. 2B). Colony formation assays indicated that the clone numbers and colony size was attenuated in the HNF1A-AS1 knockdown group, suggesting that depletion of HNF1A-AS1 slow down the growth of GBM cells (Fig. 2C). Wound healing assays indicated that the wound-healing capacity was worse and slower in si-HNF1A-AS1 groups than in si-NC groups (Fig. 2D). In addition, transwell assay showed that knockdown of HNF1A-AS1 significantly reduced migratory and invasive capacity compared with si-NC groups (Fig. 2E). On the contrary, overexpression of HNF1A-AS1 obviously promote the malignancy of GBM cells (Fig. S1A–C). To sum up, these results proved that HNF1A-AS1 plays an important role in promoting GBM cells malignant behaviors.

### EGR1 strengthen HNF1A-AS1 expression in GBM cells

**Fig. 3:**
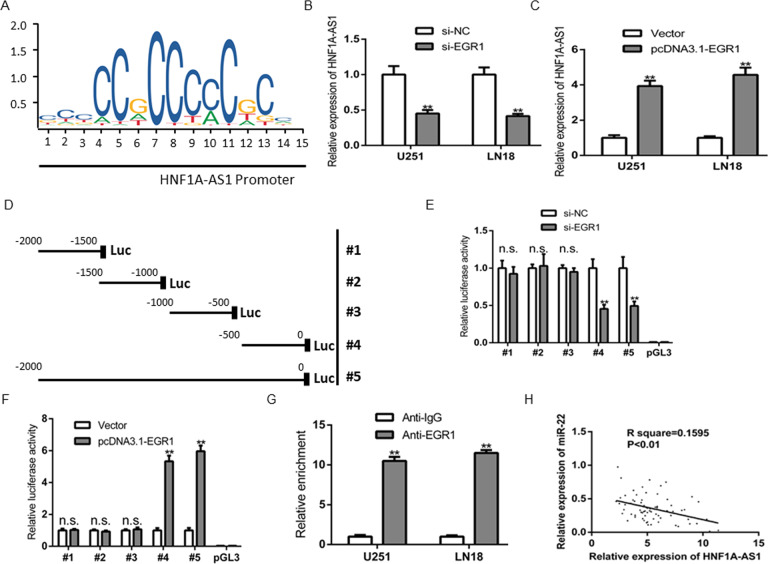
The transcription factor EGR1, specially activate HNF1A-AS1 expression at transcriptional level. **A** JASPAR database was adopted to predict the putative bind site of EGR1 on the promoter region of HNF1A-AS1. **B**, **C** The expression level of HNF1A-AS1 was tested in GBM cells transfected with si-EGR1 or pcDNA3.1-EGR1 using RT-QPCR assay. **P < 0.01 vs. si-NC or **P < 0.01 vs. Vector. **D** Serial truncations of HNF1A-AS1 promoter fragments spanning from −2000/ −1500/ −1000/ −500/ to 0. **E**, **F** This promoter fragments were cloned into pGL3-basic vectors, and dual luciferase reporter assays were performed to assess the exact EGR1 binding site on the HNF1A-AS1 promoter region. **P < 0.01 vs. si-NC or **P < 0.01 vs. Vector, n.s. indicates no significance relative to si-NC or Vector. **G** ChIP assay showed that the binding affinity between EGR1 and HNF1A-AS1 promoter region. **P < 0.01 vs. IgG. **H** Pearson’s correlation was performed to analyze the relationship between HNF1A-AS1 expression and miR-22 expression in 72 GBM patients.

We next discover the potential regulatory mechanisms which cause the upregulation of HNF1A-AS1 in GBM. By using UCSC Genome Browser, we found that Early Growth Response gene 1 (EGR1) is a latent transcription factor of HNF1A-AS1. Furthermore, previous study has demonstrated that EGR1 transcriptionally activated HNF1A-AS1 in human gastric cancer [25]. According to JASPAR database, the predicted transcription factor-binding site of EGR1 in the HNF1A-AS1 promoter is indicated in (Fig. 3A). Importantly, TCGA database confirmed that EGR1 is highly expressed in GBM tissues, and patients with higher HNF1A-AS1 expression indicated a shorter survival time (Fig. S2A, B). To detect the effect of EGR1 on HNF1A-AS1 expression, we successfully establish EGR1 overexpression or knockdown model and was tested by RT-QPCR and western blot (Fig. S2C–E). As a result, depletion of EGR1 significantly decrease the levels of HNF1A-AS1, while EGR1 overexpressed dramatically increase the levels of endogenous HNF1A-AS1 (Fig. 3B, C). Luciferase reporter assay further demonstrated that the region of HNF1A-AS1 promoter is responsible for HNF1A-AS1 transcription (Fig. 3D–F). Moreover, ChIP assay showed that the EGR1 specifically associated with the promoter of HNF1A-AS1 (Fig. 3G). These results demonstrated that EGR1 enhance HNF1A-AS1 expression at transcriptional level by directly binding to its promoter.

### HNF1A-AS1 physically bound to miR-22 and induced its degradation

**Fig. 4:**
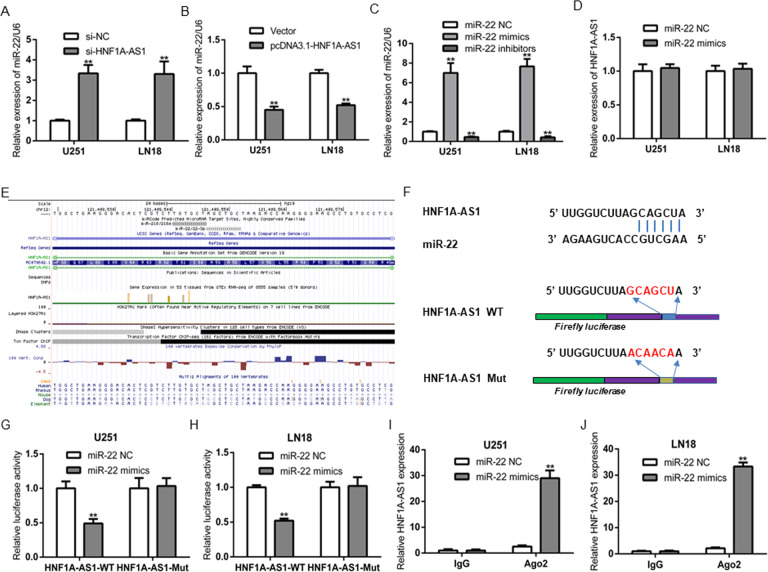
HNF1A-AS1 expression was negatively associated with miR-22 expression in GBM. **A**, **B** Relative expression of miR-22 in U251 and LN18 cells transfected with si-HNF1A-AS1, si-NC, pcDNA3.1-HNF1A-AS1 and Vector. **P < 0.01 vs. si-NC group. **C** The efficiency of miR-22 expression levels after GBM cells transfected with miR-22 NC, miR-22 mimics and miR-22 inhibitors. **P < 0.01 vs. miR-22 NC group. **D** Relative expression of HNF1A-AS1 in U251 and LN18 cells transfected with miR-22 mimics and miR-22 NC. **E**, **F** The conservation of HNF1A-AS1 in the binding site of miR-22 was snapshotted from human genome in UCSC Genome Browser and bioinformatics date predicted the putative binding sites of miR-22 on HNF1A-AS1. **G**, **H** Luciferase activity in U251 and LN18 glioma cells co-transfected with miR-22 mimics and luciferase reporters containing HNF1A-AS1-WT or HNF1A-AS1-MUT transcript. **P < 0.01 vs. miR-22 NC group. **I**, **J** RNA-IP with anti-antibody was performed in U251 and LN18 cells transfected with miR-22 NC and miR-22 mimics. HNF1A-AS1 expression level was detected using RT-QPCR. **P < 0.01 vs. miR-22 NC group. Data were presented as mean ± SD from three independent experiments.

To further clarify the underlying molecular mechanism by which HNF1A-AS1 regulates GBM cells proliferation, migration and invasion. As we all know, lncRNAs could restrain miRNAs expression and activity on their target mRNAs, by function as a ceRNA [26]. Bioinformatics data such as RegRNA 2.0, Diana-lncBase and miRANDA, were performed to predict the potential miRNA targets of HNF1A-AS1. According to the mirSVR and PhastCons scores, we found that miR-22 contain the potential target sites on HNF1A-AS1. Our studies indicated that the expression of miR-22 was significantly inverse in correlation with HNF1A-AS1 in GBM tissues (Fig. 3H). Moreover, HNF1A-AS1 knockdown dramatically increased miR-22 expression, while GBM cells transfected with PCDNA3.1-HNF1A-AS1 significantly inhibited the expression of miR-22 (Fig. 4A, B). However, no expression changed on HNF1A-AS1 when GBM cells transfected with miR-22 mimics or inhibitors (Fig. 4C, D). These results indicated that miR-22 was negatively regulated by HNF1A-AS1 in GBM.

Bioinformatics analysis predicted the potential binding sites of miR-22 on HNF1A-AS1 and the conservation of HNF1A-AS1 in the binding site of miR-22 was snapshotted from human genome in UCSC Genome Browser (Fig. 4E, F). Then, luciferase reporter assay indicated that there was no significant difference in the relative luciferase activity between HNF1A-AS1-Mut+ miR-22 mimics and HNF1A-AS1-Mut+ miR-22 NC groups, but co-transfection of pmirGLO-HNF1A-AS1-WT and miR-22 mimics dramatically reduced the luciferase activity compared with HNF1A-AS1-WT + miR-22 NC groups (Fig. 4G, H). To confirm whether HNF1A-AS1 and miR-22 are in the same RNA-induced silencing complex (RISC), we conducted anti-Ago2 RNA-binding protein immunoprecipitation (RIP) assay, and the results showed that Ago2 antibody enriched HNF1A-AS1 (Fig. 4I, J). These findings suggested that HNF1A-AS1 directly targeted miR-22 in GBM cells.

### Downregulation of miR-22 promotes GBM cells malignant behaviors

**Fig. 5:**
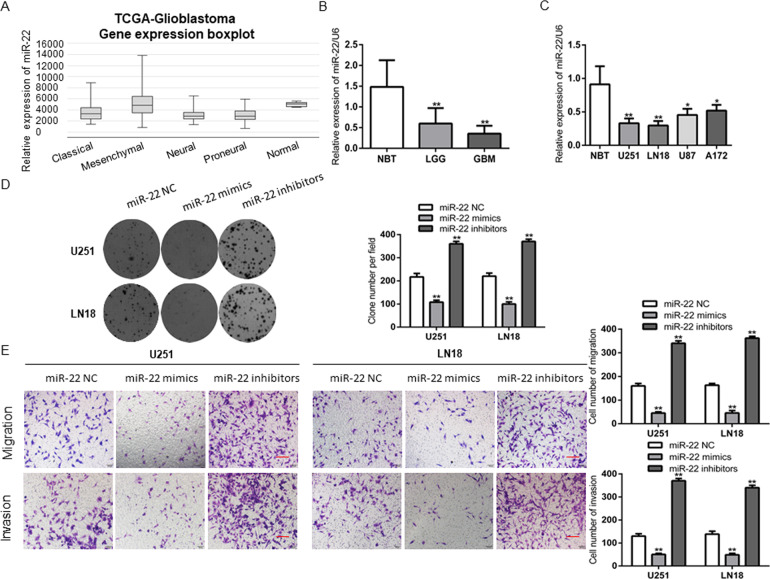
MiR-22 was downregulated in GBM. **A**, **B** TCGA date indicated that miR-22 was lowly expressed in GBM and was inversely correlated with pathological grades of glioma. **P < 0.01 vs. NBTs group. **C** RT-QPCR analysis the expression levels of miR-22 in normal human astrocyte (HA) and four GBM cell lines (U251, LN18, U87 and A172). *P < 0.05, **P < 0.01 vs. HA group. **D**, **E** CCK-8 assay and Transwell assay was performed to determine the proliferation, migration and invasion effect of miR-22 mimics and miR-22 inhibitors after transfected with U251 and LN18 cells (scale bar: 200 μm for Transwell assay). **P < 0.01 vs. miR-22 NC.

According to the TCGA data, the expression of miR-22 was obviously decreased in GBM samples, and was negatively correlated the pathological grades of glioma (Fig. 5A), which was coincided with our study (Fig. 5B). RT–QPCR analysis confirmed that miR-22 expression was significantly downregulated in four GBM cell lines (Fig. 5C). Compared with the miR-22 NC group, the proliferation, migration and invasion ability of GBM cells were distinctly reduced when cells transfected with miR-22 mimics, and cells’ malignant behavior ability were enhanced when cells transfected with miR-22 inhibitors (Fig. 5D–E). These results indicated that miR-22 acted as an anti-oncogene in GBM cells.

### ENO1, a direct target of miR-22 in GBM cells

**Fig. 6:**
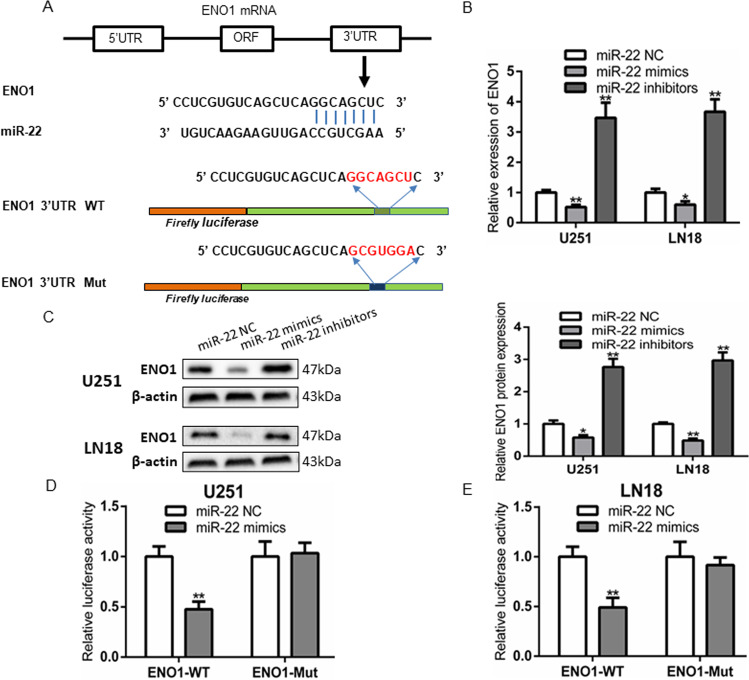
MiR-22 targets the 3’UTR of ENO1 and inhibits its expression in GBM. **A** Schematic diagram showing the predicted miR-22 binding sites within the 3’UTR of oncogene ENO1. B, **C** Relative expression of ENO1 mRNA and protein levels in U251 and LN18 cells after transfected with miR-22 mimics, miR-22 inhibitors, and miR-22 NC. *P < 0.05, **P < 0.01 vs. miR-22 NC group. **D**, **E** Luciferase activity in U251 and LN18 cells co-transfected with miR-22 mimics and luciferase reporters containing ENO1 wild type (WT) or mutant type (MUT) 3′-UTR. **P < 0.01 vs. miR-22 NC group. Data are presented as mean ± SD from three independent experiments.

Bioinformatic tools were adopted to predict potential targets of miR-22 in GBM cells. We found that 3′UTR regions of the overlapped potential candidates both have the predicted binding sites of miR-22. Ultimately, a key glycolytic enzyme, ENO1, was identified as target gene, in view of its upregulation is associated with glioma progression and prognosis [27, 28]. A previous study showed that miR-22 suppresses the proliferation of retinoblastoma cells by inhibiting ENO1, and ENO1 was a target of miR-22 [29]. As showed in Fig. 6A, the presumptive binding sites of miR-22 within the 3’UTR of ENO1 were predicted by TargetScan and MirDB. To confirm whether miR-22 regulated ENO1, GBM cells were transfected with miR-22 mimic or miR-22 inhibitors. The results indicated that increasing miR-22 markedly suppressed ENO1 mRNA and protein levels compared to miR-22 NC and conversely ENO1 expression significantly increased after inhibited miR-22 (Fig. 6B, C). Then, luciferase reporter assay demonstrated ed that co-transfection of ENO1 WT and miR-22 mimics drastically reduced luciferase activity compared with the ENO1 WT + miR-22 NC group, whereas miR-22 Mut binding site within ENO1 abrogated the inhibitory effect of miR-22 mimics on the reporter gene expression (Fig. 6D, E). In addition, we found that ENO1 was significantly overexpressed in GBM tissues and its high expression was inverse correlation with overall survival time in GBM TCGA data, which was consistent with CGGA data (Fig. S3A–D). These findings indicated that miR-22 inhibited ENO1 expression in GBM cells by targeting the 3′ UTR of oncogene ENO1.

### Knockdown of HNF1A-AS1 induced suppression of malignant phenotype was smothered by knockdown of miR-22 in GBM cells

**Fig. 7:**
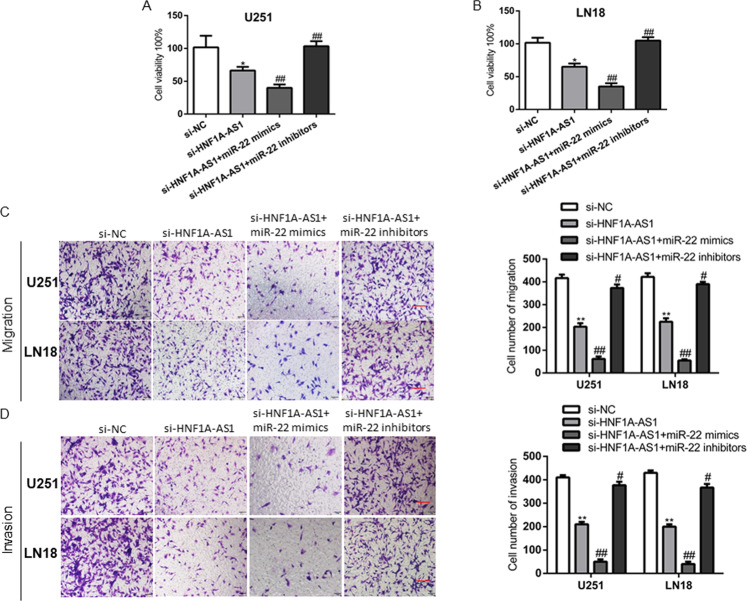
MiR-22 inhibition reversed the si-HNF1A-AS1 induced inhibitory effects on GBM cells. **A**, **B** CCK-8 assay was performed to determine the proliferation after co-transfected with si-HNF1A-AS1 and miR-22 mimics or miR-22 inhibitors. *P < 0.05 vs. si-NC group; ##*P* < 0.01 vs. si-HNF1A-AS1 group. **C**, **D** Transwell assay in U251 and LN18 cells was performed to determined cell migration and invasion ability after co-transfected with si-HNF1A-AS1 and miR-22 mimics or miR-22 inhibitors (scale bar: 200 μm). **P < 0.01 vs. si-NC group; #*P* < 0.05, ##*P* < 0.01 vs. si-HNF1A-AS1 group. Data are presented as mean ± SD from three independent experiments.

Previous study has confirmed that ENO1 attenuated led to underpin cancer progression in glioma cells [27]. However, whether HNF1A-AS1 promote the malignant behaviors of GBM cells by inhibiting miR-22 remained largely unknown. miR-22 mimics and miR-22 inhibitors were transfected into si-HNF1A-AS1 GBM cells. The results indicated that cell proliferation, migration and invasion ability were attenuated in si-HNF1A-AS1 and miR-22 mimics groups. si-HNF1A-AS1 combined with miR-22 mimics group was strongly reduced the malignant phenotype of GBM cells, while miR-22 inhibitors reversed the suppression of HNF1A-AS1 attenuated in GBM cells (Fig. 7A–D). Therefore, these results suggested that HNF1A-AS1 exerts its biofunctional roles though miR-22 in GBM cells.

### HNF1A-AS1 functions as a ceRNA for ENO1 via modulating miR-22 and advances tumorigenesis in vivo in GBM

**Fig. 8:**
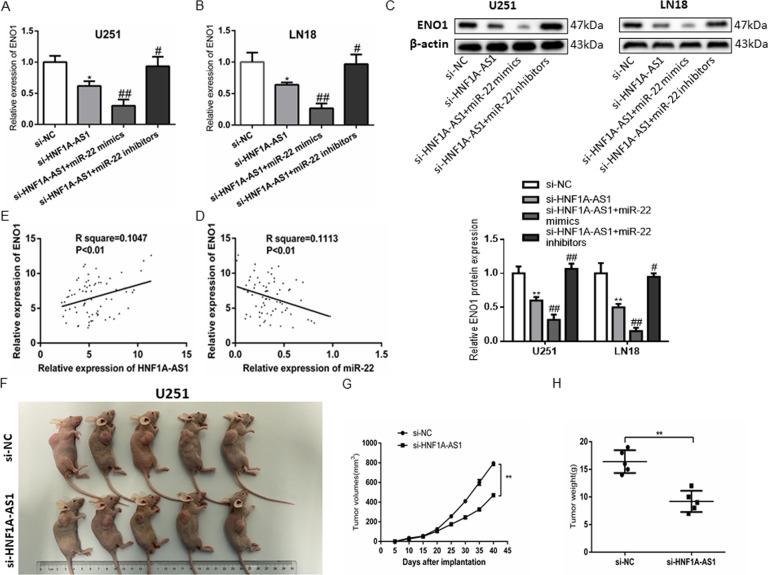
HNF1A-AS1 strengthen ENO1 expression by inhibiting miR-22 in GBM, and promotes tumor growth in vivo. **A**–**C** RT-QPCR and Western blot assays were performed to detect the mRNA and protein levels of ENO1 after cells transfected with si-HNF1A-AS1 and miR-22 mimics or miR-22 inhibitors. *P < 0.05, **P < 0.01 vs. si-NC group; #*P* < 0.05, ##*P* < 0.01 vs. si-HNF1A-AS1 group. Data are presented as mean ± SD from three independent experiments. **D** Pearson’s correlation analysis of the relationship between ENO1 expression and HNF1A-AS1 expression. **E** Pearson’s correlation analysis of the relationship between ENO1 expression and miR-22 expression. **F** U251 cells were stably transfected with si-NC or si-HNF1A-AS1, which were injected subcutaneously into nude mice, respectively. **G** Tumor volumes were calculated every 5 days after injection. **P < 0.01 vs. si-NC group. **H** Tumor weight were significantly decreased in the si-HNF1A-AS1 group. **P < 0.01 vs. si-NC group.

To explore whether HNF1A-AS1 regulates the expression of ENO1 by inhibiting miR-22 in GBM. ENO1 mRNA and protein levels were detected by RT-QPCR and Western blot assays after U251 and LN18 cells co-transfected with si-HNF1A-AS1 and miR-22 mimics or miR-22 inhibitors. The results showed that HNF1A-AS1 knockdown decreased the mRNA and protein levels of ENO1 compare with si-NC, and si-HNF1A-AS1 combined with miR-22 mimics drastically decreased the expression levels of ENO1 but were reversed by co-transfection with si-HNF1A-AS1 and miR-22 inhibitors (Fig. 8A–C). Furthermore, our study first found that ENO1 expression was negatively correlated to miR-22 and positively correlated to HNF1A-AS1 in 72 GBM patients (Fig. 8D, E). Hence, these results indicated that HNF1A-AS1 regulates ENO1 expression by sponging miR-22 in GBM.

Tumor xenograft models were performed to evaluate the functional roles of HNF1A-AS1 in vivo. We inoculated subcutaneously the treated U251 cell, as showed in (Fig. 8A–C), Tumor volumes and tumor weights in the si-HNF1A-AS1 group were obviously smaller and lower compared with the si-NC group. Also, western blot indicated that ENO1 protein expression levels in nude mice tumor tissues was significantly decreased in the si-HNF1A-AS1 group than in the si-NC group (Fig. S4).

## Discussion

GBM is the most common and lethal primary malignant brain cancer in adults [30]. Although Maximal safe resection and chemoradiotherapy are known as comprehensive regimens which are the most popular mean for the treatment of patients with GBM, the overall survival time of GBM patients is still poor [31, 32]. Therefore, the diagnosis, treatment and prognosis estimation of such brain tumors remain a challenge in our clinical work. What is encouraging is that the research of molecular diagnosis participated in the progression of glioma such as IDH, TP53, EGFR, H3K27M and WNT, has gain much progress [33–37].

Recently, accumulating studies have confirmed that dysregulation of lncRNAs play a considerable functional role in carcinogenesis and progression of multiple cancers. Moreover, a number of studies have demonstrated that lncRNAs could serve as a diagnostic biomarker and therapeutic target in GBM [38]. Previous studies have confirmed that lncRNAs are dysregulated in GBM. For instance, NEAT1, a glioblastoma-associated lncRNA, was an oncogenic factor that was regulated by EGFR pathway, by activating WNT/β-Catenin pathway to promote GBM cells growth and invasion [39]. Our group’s previous research found that ATB was associated with a poor clinical outcome in glioma patients, and its depletion attenuated glioma biological characteristics by directly repression miR-200a, and positively regulating TGF-β2 expression in glioma cells [40].

In this study, we found HNF1A-AS1 was significantly upregulated in GBM tissues and cell lines compared with normal brain tissues and HA cell. High expression of HNF1A-AS1 was negatively associated with the clinical outcomes of GBM patients. In addition, knockdown of HNF1A-AS1 inhibited cell proliferation, colony formation, migration and invasion in vitro and in vivo, while overexpression HNF1A-AS1 strengthen the malignant behaviors of GBM cells. Taken together, our results indicated that HNF1A-AS1 functions as an oncogene and may serve as a potential prognostic biomarker in GBM.

Mounting studies have demonstrated that lncRNAs expression could be activated by their upstream transcription factors. For instance, MKL1 induce the transactivation of SNHG18, which promotes NSCLC growth, invasion, and metastasis [41]. The transcription factor TEAD4 mediates MNX1-AS1 expression to drive gastric cancer progression [42]. EGR1 as a transcription activator several lncRNAs [43, 44]. In the present study, we found that EGR1 could bind with HNF1A-AS1 promoter region and transcriptionally induced HNF1A-AS1 overexpression in GBM cells, and TCGA datasets confirmed upregulated EGR1 is correlated with poor prognosis of GBM patients.

Recently, increasing evidences demonstrated that lncRNAs can competitively binding to miRNAs and then regulate the expression of miRNA downstream target genes [8]. Liu et al. find that high HNF1A-AS1 expression function as a ceRNA that sponging miR-661, thereby increasing CDC34 and in turn accelerating HNF1A-AS1 expression in in gastric cancer [25]. Cai et ai. show that HNF1A-AS1 is overexpressed in colon tissues and cell lines, and served as a ceRNA to modulate miRNA-34a expression, subsequently with repression of miR-34a/SIRT1/p53 feedback loop and activation of canonical Wnt signaling pathway in metastasis of colon cancer [45]. Here, we found that HNF1A-AS1 located in both the nucleus and cytoplasm, indicating its complicated functions. To clarify the underlying mechanism by which HNF1A-AS1 functions as an oncogene in GBM. According to our bioinformatics analysis, we found that miR-22 might has putative binding sites with HNF1A-AS1 in GBM. Moreover, a negative association between HNF1A-AS1 and miR-22 expression in GBM tissues was confirmed from our study. HNF1A-AS1 knockdown or overexpression significantly increased or decreased the expression of miR-22 in GBM cells. In addition, dual-luciferase reporter and RIP assay demonstrated that HNF1A-AS1 acted as miRNA sponge and negatively regulates miR-22 expression in GBM cells.

MiR-22, which was an exon of the C17orf91 gene, was located at chromosome 17p13.3. Previous studies have confirmed that miR-22 is markedly downregulated and functions as a tumor suppressor miRNA in various cancers. Sun et al. find that miR-22 is downregulated in colon cancer, overexpression of miR-22 significantly inhibits cell proliferation, migration, metastasis, and epithelial-mesenchymal (EMT) transition by directly targeting BCL9L [46]. Jiang et al. show that miR-22 is significantly downregulated in AML and forced expression of miR-22 significantly suppresses leukemic cell viability and growth, and restoration of miR-22 expression holds great therapeutic potential to treat AML [47]. Chen et al. find that miR-22 mimics suppresses cell proliferation, migration, and invasion via targeting the 3′-UTR of SIRT1 in the progression of GBM [48]. However, many other important downstream target genes of miR-22 in GBM are not clear. In our study, we found miR-22 expression was downregulated in GBM tissues and cells in comparison with normal tumor tissues and HA cell. Furthermore, overexpression of miR-22 remarkably suppressed cell proliferation, migration, and invasion, and miR-22 inhibition exhibited the opposite effects. In addition, miR-22 suppression reversed the inhibitory effects caused by HNF1A-AS1 knockdown. In a word, these findings suggested that HNF1A-AS1 aggravated the biological characteristic of GBM cells by directly targets miR-22.

Alpha-enolase (ENO1), a famous glycolytic enzyme functioning during aerobic glycolysis, was found in nearly all parts of adult human, and contributed to the Warburg effect in cancer cells [49]. Previous studies showed that ENO1 was up-regulated and functioned as an oncogene in various cancer types [50, 51]. Song et al. find that elevated ENO1 expression was an independent prognostic factor, and boosted cell proliferation, migration, and invasion ability by activating the PI3K/AKT pathway in glioma cells [27]. Principe et al. show that ENO1 silencing which increased integrins and uPAR (an ECM receptor), could impeded cell adhesion, invasion, and metastasis, by acting as a plasminogen receptor on the tumor cell surface in pancreatic cancer [52]. Fu et al also demonstrate that upregulated ENO1 drastically enhanced NSCLC cell glycolysis and malignant biological behaviors by activating FAK-mediated PI3K/AKT pathway and its downstream signals to regulate the glycolysis, cell cycle, and EMT-associated genes [53]. In our study, we found both TCGA data and CGGA data validated that ENO1 was upregulated in GBM tissues and high ENO1 expression indicated a poor outcome of GBM patients. We confirmed that miR-22 directly target the 3′UTR of ENO1 and negatively regulate its expression. More importantly, we further validated that HNF1A-AS1 knockdown induced the reduction of ENO1 expression was regain via miR-22 inhibition, indicating HNF1A-AS1 could positively regulate ENO1 expression by inhibiting miR-22 expression in GBM. These results suggested that HNF1A-AS1 functioned as a ceRNA by competitively binding miR-22 and releasing ENO1 in GBM.

In conclusion, our data demonstrate that a crucial oncogenic transcription factor EGR1 mediates HNF1A-AS1 expression via binding to the promoter region of HNF1A-AS1, which is highly expressed in GBM tissues and cell lines, and was negatively correlated with GBM progression and prognosis. HNF1A-AS1 suppressed GBM malignancy by functioning as a ceRNA to sponge miR-22 and facilitates the expression of ENO1, which is a direct target of miR-22 in GBM. Therefore, our findings support that HNF1A-AS1 may become a new therapeutic target for GBM treatment.

## Materials and methods

### Patient samples

72 GBM samples and 41 low-grade glioma (LGG) tissues were derived from surgical excision, and 15 normal brain tissues (NBT) come from acute brain injury patients during surgical. All patient samples were obtained from the Department of Neurosurgery, the Second Affiliated Hospital of AnHui Medical University from January 2013 to October 2018. All patients signed the written informed consent, and simultaneously approved by the Clinical Research Ethics Committee at the Second Affiliated Hospital of AnHui Medical University.

### Bioinformatics data

UCSC Genome Browser (http://genome.ucsc.edu/), JASPAR (http://jaspar.genereg.net/), The Cancer Genome Atlas (TCGA, http://cancergenome), RegRNA 2.0 (http://regrna2.mbc.nctu.edu.tw/), Diana-lncBase (http://carolina.imis.athena-innovation.gr), miRANDA (http://www.microrna.org), Chinese Glioma Genome Atlas (CGGA, www.cgga.org), TargetScan (http://www.targetscan.org), MirDB (http://www.mirdb.org/).

### Cell culture

Human GBM cell lines (U251, LN18, U87 and A172), normal human astrocytes (HA) were purchased from the American Type Culture Collection (ATCC). All cells were cultured in DMEM with 10% FBS and streptomycin (100 μg/ml), penicillin (100 U/ml). All cell lines were cultured at 37 °C in a humidified incubator with 5 % CO2.

### Subcellular fractionation

Cytoplasmic and nuclear fractions of the LN18 cells were prepared and collected according to the manufacturer’s instructions of PARIS™ Kit (Life Technologies, CA, USA). GAPDH was used as the cytoplasmic internal reference. U6 small nuclear RNA was used as the nuclear the internal reference.

### Cell transfection

HNF1A-AS1 siRNA (si-HNF1A-AS1), EGR1 siRNA (si-EGR1) and corresponding negative control (si-NC) were synthesized by Guangzhou RiboBio Co., Ltd. (Guangzhou, China). The sequence of si-HNF1A-AS1-1 was as follows: CCCTCCATCTAACATTCAA, si-EGR1, CAACGAGAAGGTGCTGGTG. U251 and LN18 cells transfected with these fragments respectively by using Lipofectamine3000 (Invitrogen, USA). Full‐length of HNF1A‐AS1 and EGR1 was amplified by PCR and sub-cloned into pcDNA3.1-Vector (pcDNA3.1-HNF1A-AS1 and Vector), pcDNA3.1-Vector (pcDNA3.1-EGR1 and Vector) (Sangon Biotech, Shanghai, China). MiR-22-3p negative control (NC), miR-22-3p mimics and miR-22-3p inhibitors were purchased from Guangzhou RiboBio (Guangzhou, China).

### RT–QPCR

Cells and tissues were lysed in TRIzol (Invitrogen, USA), and Total RNA isolation was reverse transcribed to complementary DNA (cDNA) using the PrimeScript RT (Takara, Nanjing, China). The primers for genes were determined as follows: HNF1A-AS1 forward 5′- CAAGAAATGGTGGCTATGA-3′, reverse 5′- TGGACTGAAGGACAAGGGT-3′; GAPDH forward 5′-AGCAAGAGCACAAGAGGAAG-3′, reverse 5′-GGTTGAGCACAGGGTACTTT-3′.

EGR1, forward: 5’-CAGCACCTTCAACCCTCAG-3′, reverse: 5’-CACAAGGTGTTGCCACTGTT-3′; ENO1 forward 5′- GCCTCCTGCTCAAAGTCAAC-3′, reverse 5′- AACGATGAGACACCATGACG-3′; GAPDH and U6 were used as loading control for HNF1A-AS1, ENO1 and miR-22. All RT–QPCR reactions were performed in triplicate. The data were determined using the 2^−△△Ct^ method.

### Cell proliferation assay

U251and LN18 cells after transfection were placed into 96-well plate (1000 cells/well), and cultured them at 37 °C with five percent CO2. Approximately 10 μl of CCK (Dojindo, Shanghai, China). solution was added into per well. Finally, the absorbance at 450 nm was measured using a ST-360 micro-plate reader (KHB, Shanghai, China) after incubated at 37 °C for 2 h.

### Colony formation assay

For the clone formation assay, 48 h after transfection, GBM cells (200 viable cells per well) were seeded in a 6-well plate and cultured with complete medium for 12 days. cells were fixed with 4 % polyoxymethylene and stained with 1.5 % methylene blue for 30 min at room temperature.

### Wound healing

U251and LN18 cells after transfection 48 h were seeded in a 6-well plate and cultured with complete medium (200 viable cells per well). a 10-μl pipette tip was used to create wound gaps, gently washed, and cultured with serum-free medium for 24 h. The wound gaps were observed at 0 and 24 h after wounding and photographed with a light microscope (Olympus, Japan).

### Transwell assay

The 24-well chambers with 8 μm polycarbonate membrane inserts (Corning, New York, USA) was used to detect the migration and invasion ability. A total of 2×10^4^ cells were resuspended in 150 μl serum‑free medium and 500 μl of 10 % FBS medium, respectively place in the upper chamber with or without pre-coated with 400 ng/ml Matrigel solution (BD Biosciences, New jersey, USA), and placed in the lower chamber of Transwell plates. After 48 h, the migrated and invaded cells on the lower chamber membrane were fixed with 4% polyoxymethylene and stained with crystal violet (Sigma). Five predetermined fields were counted under a microscope (Olympus, Japan). All assays were performed in triplicate.

### Luciferase reporter assays

The fragments of HNF1A-AS1 and 3′ UTR of ENO1, both containing the predicted miR-22 binding site, then the predicted wild-type (WT) binding sites of miR-22 and mutant binding sites (Mut) were cloned into a pmiRGLO Dual-luciferase miRNA Target Expression Vector (Promega, Madison, WI, USA), termed as pmiRGLO-HNF1A-AS1-wild-type (HNF1A-AS1-WT), pmiRGLO-HNF1A-AS1-mutated-type (HNF1A-AS1-Mut), pmiRGLO-ENO1-wild-type (ENO1-WT) and pmiRGLO-ENO1-mutated-type (ENO1-Mut). Then HNF1A-AS1-WT or HNF1A-AS1-Mut was co-transfected with the miR-22 negative control or mimics into GBM cells by using Lipofectamie3000 (Invitrogen, USA). Dual-Luciferase Reporter Assay System (Promega, Madison, WI, USA) was used to detect the relative luciferase activity. ENO1-WT and ENO1-Mut were handled similarly as described above.

To confirm the bind relation between EGR1 and HNF1A-AS1 promoter, pGL3-HNF1A-AS1 promoter was co-transfected into cells along with si-EGR1 or si-NC using Lipofectamine3000. The luciferase activity was measured by a Dual-Luciferase reporter assay system (Promega, USA). All assays were independently performed in triplicate.

### Chromatin immunoprecipitation assay

The EZ-Magna ChIP™ Chromatin Immunoprecipitation Kit (Millipore, USA) was used for Chromatin immunoprecipitation (ChIP) assay. U251 and LN18 cells were fixed with 1% formaldehyde for 10 min at room temperature and lysed in ChIP lysis buffer, and then the DNA was sonicated for shearing DNA into 500-bp fragments. Subsequently, DNA samples were precipitated with anti-IgG or anti-EGR1 antibody and Protein A/G magnetic beads for overnight. Finally, the co-precipitated chromatin DNA was collected, and was tested by RT-QPCR.

### RNA immunoprecipitation

RNA immunoprecipitation (RIP) experiments were performed by the Magna RIP™ RNA-Binding Protein Immunoprecipitation Kit (Millipore, USA), and was conducted as previously described [40].

### Western blotting

Protein extract from U251, LN18 cells and tissue samples by using RIPA protein extraction reagent (Beyotime, Shanghai, China). The concentration of protein was tested by the BCA Protein Assay Kit (Beyotime, Shanghai, China). Then on ice, subjected to SDS-PAGE and transferred to PVDF membranes (Millipore, USA), which were blocked in buffer (5% nonfat milk in TBST) about 1.5 h before being incubated with primary antibodies (anti-rabbit-ENO1, 1:1000, Abcam, EUGENE, USA), (anti-rabbit-EGR1, 1:1000, CST, USA) and anti-rabbit-β-actin (1:1000, Abcam, EUGENE, USA) at 4 °C overnight. Horseradish peroxidase-conjugated goat anti-rabbit (1:5000, Beyotime, Shanghai, China) was applied as a secondary antibody and incubated at 4 °C for 1 h, and then immune complexes were visualized by ECL reagent.

### In vivo xenograft model

Female nude mice at 4–6 weeks of age were used in this study, and were divided into two groups (five/group). U251 cells stably transfected with si-NC or si-HNF1A-AS1 were collected, and injected into the subcutaneous tissues of the axillary skin. The growth of tumor was measured every five days. 40 days after injection, the mice were sacrificed, and the tumor nodules were harvested for further study. All experiments was was approved by the Animal Care and Use Committee of AnHui Medical University.

### Statistical analysis

Unless stated otherwise, all experiments were performed in triplicate and all data were presented as the mean ± standard deviation (SD). GraphPad Prism V6.01 (GraphPad Software, Inc., La Jolla, CA, USA) software was used for statistical analysis and generate figures. Differences were analyzed by SPSS statistical software (version 19.0, Armonk, NY, USA) with the Student’s t-test or one-way ANOVA. Pearson’s correlation was performed to analyze the relationship between the expression of HNF1A-AS1, miR-22 and ENO1 in tissues. Survival analysis was performed using the Kaplan-Meier method and log-rank tests in GraphPad Prism 6.01. Differences were considered significant if *P* < 0.05.

## Supplementary information

Supplementary figure legends

Figure S1

Figure S2

Figure S3

Figure S4

Attribution of Authorship

## Data Availability

Data supporting present findings are available from the corresponding author upon reasonable request.

## References

[1] Ostrom QT, Gittleman H, Liao P, Vecchione-Koval T, Wolinsky Y, Kruchko C (2017). CBTRUS Statistical Report: Primary brain and other central nervous system tumors diagnosed in the United States in 2010-2014. Neuro Oncol.

[2] Lieberman F (2017). Glioblastoma update: molecular biology, diagnosis, treatment, response assessment, and translational clinical trials. F1000Res.

[3] Reifenberger G, Wirsching HG, Knobbe-Thomsen CB, Weller M (2017). Advances in the molecular genetics of gliomas - implications for classification and therapy. Nat Rev Clin Oncol.

[4] Delgado-Martin B, Medina MA (2020). Advances in the knowledge of the molecular biology of glioblastoma and its impact in patient diagnosis, stratification, and treatment. Adv Sci.

[5] Gandhi M, Gross M, Holler JM, Coggins SA, Patil N, Leupold JH (2020). The lncRNA lincNMR regulates nucleotide metabolism via a YBX1 - RRM2 axis in cancer. Nat Commun.

[6] Dong Z, Gao M, Li C, Xu M, Liu S (2020). LncRNA UCA1 antagonizes arsenic-induced cell cycle arrest through destabilizing EZH2 and facilitating NFATc2 expression. Adv Sci.

[7] Jin X, Ge LP, Li DQ, Shao ZM, Di GH, Xu XE (2020). LncRNA TROJAN promotes proliferation and resistance to CDK4/6 inhibitor via CDK2 transcriptional activation in ER+ breast cancer. Mol Cancer.

[8] Liang Y, Song X, Li Y, Chen B, Zhao W, Wang L (2020). LncRNA BCRT1 promotes breast cancer progression by targeting miR-1303/PTBP3 axis. Mol Cancer.

[9] Liu J, Liu ZX, Wu QN, Lu YX, Wong CW, Miao L (2020). Long noncoding RNA AGPG regulates PFKFB3-mediated tumor glycolytic reprogramming. Nat Commun.

[10] Zheng S, Yang L, Zou Y, Liang JY, Liu P, Gao G (2020). Long non-coding RNA HUMT hypomethylation promotes lymphangiogenesis and metastasis via activating FOXK1 transcription in triple-negative breast cancer. J Hematol Oncol.

[11] Li Z, Zhang J, Liu X, Li S, Wang Q, Di C (2018). The LINC01138 drives malignancies via activating arginine methyltransferase 5 in hepatocellular carcinoma. Nat Commun.

[12] Yari H, Jin L, Teng L, Wang Y, Wu Y, Liu GZ (2019). LncRNA REG1CP promotes tumorigenesis through an enhancer complex to recruit FANCJ helicase for REG3A transcription. Nat Commun.

[13] Li D, Liu X, Zhou J, Hu J, Zhang D, Liu J (2017). Long noncoding RNA HULC modulates the phosphorylation of YB-1 through serving as a scaffold of extracellular signal-regulated kinase and YB-1 to enhance hepatocarcinogenesis. Hepatology.

[14] Ji J, Xu R, Ding K, Bao G, Zhang X, Huang B (2019). Long noncoding RNA SChLAP1 forms a growth-promoting complex with HNRNPL in human glioblastoma through stabilization of ACTN4 and activation of NF-kappaB signaling. Clin. Cancer Res.

[15] Tang F, Wang H, Chen E, Bian E, Xu Y, Ji X (2019). LncRNA-ATB promotes TGF-beta-induced glioma cells invasion through NF-kappaB and P38/MAPK pathway. J Cell Physiol.

[16] Xu J, Yang B, Wang L, Zhu Y, Zhu X, Xia Z, et al. LncRNA BBOX1-AS1 upregulates HOXC6 expression through miR-361-3p and HuR to drive cervical cancer progression. Cell Prolif. e12823 (2020). 10.1111/cpr.12823 PMC7377938 32515533

[17] Liang Y, Chen X, Wu Y, Li J, Zhang S, Wang K (2018). LncRNA CASC9 promotes esophageal squamous cell carcinoma metastasis through upregulating LAMC2 expression by interacting with the CREB-binding protein. Cell Death Differ.

[18] Wu W, Yu T, Wu Y, Tian W, Zhang J, Wang Y (2019). The miR155HG/miR-185/ANXA2 loop contributes to glioblastoma growth and progression. J Exp Clin Cancer Res.

[19] Ren S, Xu Y (2019). AC016405.3, a novel long noncoding RNA, acts as a tumor suppressor through modulation of TET2 by microRNA-19a-5p sponging in glioblastoma. Cancer Sci..

[20] Li Q, Dong C, Cui J, Wang Y, Hong X (2018). Over-expressed lncRNA HOTAIRM1 promotes tumor growth and invasion through up-regulating HOXA1 and sequestering G9a/EZH2/Dnmts away from the HOXA1 gene in glioblastoma multiforme. J Exp Clin Cancer Res.

[21] Yang X, Song JH, Cheng Y, Wu W, Bhagat T, Yu Y (2014). Long non-coding RNA HNF1A-AS1 regulates proliferation and migration in oesophageal adenocarcinoma cells. Gut.

[22] Liu Z, Li H, Fan S, Lin H, Lian W (2019). STAT3-induced upregulation of long noncoding RNA HNF1A-AS1 promotes the progression of oral squamous cell carcinoma via activating Notch signaling pathway. Cancer Biol Ther.

[23] Wang YH, Liu YH, Ji YJ, Wei Q, Gao TB (2018). Upregulation of long non-coding RNA HNF1A-AS1 is associated with poor prognosis in urothelial carcinoma of the bladder. Eur Rev Med Pharm Sci.

[24] Zhang G, An X, Zhao H, Zhang Q, Zhao H (2018). Long non-coding RNA HNF1A-AS1 promotes cell proliferation and invasion via regulating miR-17-5p in non-small cell lung cancer. Biomed Pharmacother.

[25] Liu HT, Liu S, Liu L, Ma RR, Gao P (2018). EGR1-mediated transcription of lncRNA-HNF1A-AS1 promotes cell-cycle progression in gastric cancer. Cancer Res.

[26] Feng J, Zhou Q, Yi H, Ma S, Li D, Xu Y (2019). A novel lncRNA n384546 promotes thyroid papillary cancer progression and metastasis by acting as a competing endogenous RNA of miR-145-5p to regulate AKT3. Cell Death Dis.

[27] Song Y, Luo Q, Long H, Hu Z, Que T, Zhang X (2014). Alpha-enolase as a potential cancer prognostic marker promotes cell growth, migration, and invasion in glioma. Mol Cancer.

[28] Chen S, Zhang Y, Wang H, Zeng YY, Li Z, Li ML (2018). WW domain-binding protein 2 acts as an oncogene by modulating the activity of the glycolytic enzyme ENO1 in glioma. Cell Death Dis.

[29] Liu Y, Li H, Liu Y, Zhu Z (2018). MiR-22-3p targeting alpha-enolase 1 regulates the proliferation of retinoblastoma cells. Biomed Pharmacother.

[30] Cheng X, Geng F, Pan M, Wu X, Zhong Y, Wang C, et al. Targeting DGAT1 ameliorates glioblastoma by increasing fat catabolism and oxidative stress. Cell Metab. (2020). 10.1016/j.cmet.2020.06.002 PMC7415721 32559414

[31] Pottoo FH, Javed MN, Rahman JU, Abu-Izneid T, Khan FA. Targeted delivery of miRNA based therapeuticals in the clinical management of glioblastoma multiforme. Semin Cancer Biol. (2020). 10.1016/j.semcancer.2020.04.001 32302695

[32] Turaga SM, Silver DJ, Bayik D, Paouri E, Peng S, Lauko A, et al. JAM-A functions as a female microglial tumor suppressor in glioblastoma. Neuro Oncol. 2020;22:1591–601. 10.1093/neuonc/noaa148 PMC7690368 32592484

[33] Burgenske DM, Yang J, Decker PA, Kollmeyer TM, Kosel ML, Mladek AC, et al. Molecular profiling of long-term IDH-wildtype glioblastoma survivors. Neuro Oncol. 2019;21:1458–69. 10.1093/neuonc/noz129 PMC6827834 31346613

[34] Ham SW, Jeon HY, Jin X, Kim EJ, Kim JK, Shin YJ (2019). TP53 gain-of-function mutation promotes inflammation in glioblastoma. Cell Death Differ.

[35] Guo G, Gong K, Puliyappadamba VT, Panchani N, Pan E, Mukherjee B (2019). Efficacy of EGFR plus TNF inhibition in a preclinical model of temozolomide-resistant glioblastoma. Neuro Oncol.

[36] Harutyunyan AS, Krug B, Chen H, Papillon-Cavanagh S, Zeinieh M, De Jay N (2019). H3K27M induces defective chromatin spread of PRC2-mediated repressive H3K27me2/me3 and is essential for glioma tumorigenesis. Nat Commun.

[37] Louis DN, Perry A, Reifenberger G, von Deimling A, Figarella-Branger D, Cavenee WK (2016). The 2016 World Health Organization Classification of Tumors of the Central Nervous System: a summary. Acta Neuropathol.

[38] Zong Z, Song Y, Xue Y, Ruan X, Liu X, Yang C (2019). Knockdown of LncRNA SCAMP1 suppressed malignant biological behaviours of glioma cells via modulating miR-499a-5p/LMX1A/NLRC5 pathway. J Cell Mol Med.

[39] Chen Q, Cai J, Wang Q, Wang Y, Liu M, Yang J (2018). Long noncoding RNA NEAT1, regulated by the EGFR pathway, Contributes to glioblastoma progression through the WNT/beta-catenin pathway by scaffolding EZH2. Clin Cancer Res.

[40] Ma CC, Xiong Z, Zhu GN, Wang C, Zong G, Wang HL (2016). Long non-coding RNA ATB promotes glioma malignancy by negatively regulating miR-200a. J Exp Clin Cancer Res.

[41] Fan H, Yuan J, Li Y, Jia Y, Li J, Wang X (2021). MKL1-induced lncRNA SNHG18 drives the growth and metastasis of non-small cell lung cancer via the miR-211-5p/BRD4 axis. Cell Death Dis.

[42] Shuai Y, Ma Z, Liu W, Yu T, Yan C, Jiang H (2020). TEAD4 modulated LncRNA MNX1-AS1 contributes to gastric cancer progression partly through suppressing BTG2 and activating BCL2. Mol Cancer.

[43] Lei T, Zhu X, Zhu K, Jia F, Li S (2019). EGR1-induced upregulation of lncRNA FOXD2-AS1 promotes the progression of hepatocellular carcinoma via epigenetically silencing DKK1 and activating Wnt/beta-catenin signaling pathway. Cancer Biol Ther.

[44] Ma Z, Gao X, Shuai Y, Wu X, Yan Y, Xing X (2021). EGR1-mediated linc01503 promotes cell cycle progression and tumorigenesis in gastric cancer. Cell Prolif.

[45] Fang C, Qiu S, Sun F, Li W, Wang Z, Yue B (2017). Long non-coding RNA HNF1A-AS1 mediated repression of miR-34a/SIRT1/p53 feedback loop promotes the metastatic progression of colon cancer by functioning as a competing endogenous RNA. Cancer Lett.

[46] Sun R, Liu Z, Han L, Yang Y, Wu F, Jiang Q (2019). miR-22 and miR-214 targeting BCL9L inhibit proliferation, metastasis, and epithelial-mesenchymal transition by down-regulating Wnt signaling in colon cancer. FASEB J.

[47] Jiang X, Hu C, Arnovitz S, Bugno J, Yu M, Zuo Z (2016). miR-22 has a potent anti-tumour role with therapeutic potential in acute myeloid leukaemia. Nat Commun.

[48] Chen H, Lu Q, Fei X, Shen L, Jiang D, Dai D (2016). miR-22 inhibits the proliferation, motility, and invasion of human glioblastoma cells by directly targeting SIRT1. Tumour Biol.

[49] Cairns RA, Harris IS, Mak TW (2011). Regulation of cancer cell metabolism. Nat Rev Cancer.

[50] Zhu X, Miao X, Wu Y, Li C, Guo Y, Liu Y (2015). ENO1 promotes tumor proliferation and cell adhesion mediated drug resistance (CAM-DR) in Non-Hodgkin’s Lymphomas. Exp Cell Res.

[51] Hsiao KC, Shih NY, Fang HL, Huang TS, Kuo CC, Chu PY (2013). Surface alpha-enolase promotes extracellular matrix degradation and tumor metastasis and represents a new therapeutic target. PLoS ONE.

[52] Principe M, Borgoni S, Cascione M, Chattaragada MS, Ferri-Borgogno S, Capello M (2017). Alpha-enolase (ENO1) controls alpha v/beta 3 integrin expression and regulates pancreatic cancer adhesion, invasion, and metastasis. J Hematol Oncol.

[53] Fu QF, Liu Y, Fan Y, Hua SN, Qu HY, Dong SW (2015). Alpha-enolase promotes cell glycolysis, growth, migration, and invasion in non-small cell lung cancer through FAK-mediated PI3K/AKT pathway. J Hematol Oncol.

