# Supplementary material for: EGR1 modulated LncRNA HNF1A-AS1 drives glioblastoma progression via miR-22-3p/ENO1 axis
Source: Cell Death Discov. 2021 Nov 12;7:350. doi: 10.1038/s41420-021-00734-3 (PMC8590016; doi:10.1038/s41420-021-00734-3)
Supplement: Supplementary file 1 — Supplementary figure legends [file 41420_2021_734_MOESM1_ESM.docx]

**Supplementary figure legends**

Fig. S1 Overexpression of HNF1A-AS1 enhance the malignant behaviours of GBM cells. A Relative HNF1A-AS1 expression after cells transfected with pcDNA3.1-HNF1A-AS1 or Vector. **P < 0.01 vs. Vector. B-C CCK-8 assay and Transwell assay was performed to determine the malignant behaviours ability of pcDNA3.1-HNF1A-AS1 group or Vector group (scale bar: 200 μm for Transwell assay). **P < 0.01 vs. Vector group.

Fig. S2 EGR1 is significantly increased in GBM. A-B TCGA date indicated that EGR1 was significantly upregulated in GBM, and highly EGR1 expressed indicated a poor prognosis in glioma patients. C-E Relative expression of EGR1 on mRNA and protein levels after GBM cells transfected with si-EGR1 or pcDNA3.1-EGR1 and si-NC or Vector. **P < 0.01 vs. si-NC or **P < 0.01 vs. Vector.

Fig. S3 ENO1 was obviously function as a proto-oncogene in GBM. A-B TCGA date showing that ENO1 was significantly upregulated in GBM only, not in LGG, and high ENO1 expression indicated a poor prognosis in GBM patients. C-D CGGA date further confirmed that ENO1 was significantly overexpression in GBM compared with gradeⅡglioma and grade Ⅲ glioma. Higher ENO1 expression was also associated with a poor prognosis for patients with GBM.

Fig. S4 The protein level of ENO1 in nude mice tumor tissues. Western blot tests ENO1 protein expression in nude mice tumor tissues. **P < 0.01 vs. si-NC group.
