# Supplementary figures and images for: EGR1 modulated LncRNA HNF1A-AS1 drives glioblastoma progression via miR-22-3p/ENO1 axis
Source: Cell Death Discov. 2021 Nov 12;7:350. doi: 10.1038/s41420-021-00734-3 (PMC8590016; doi:10.1038/s41420-021-00734-3)

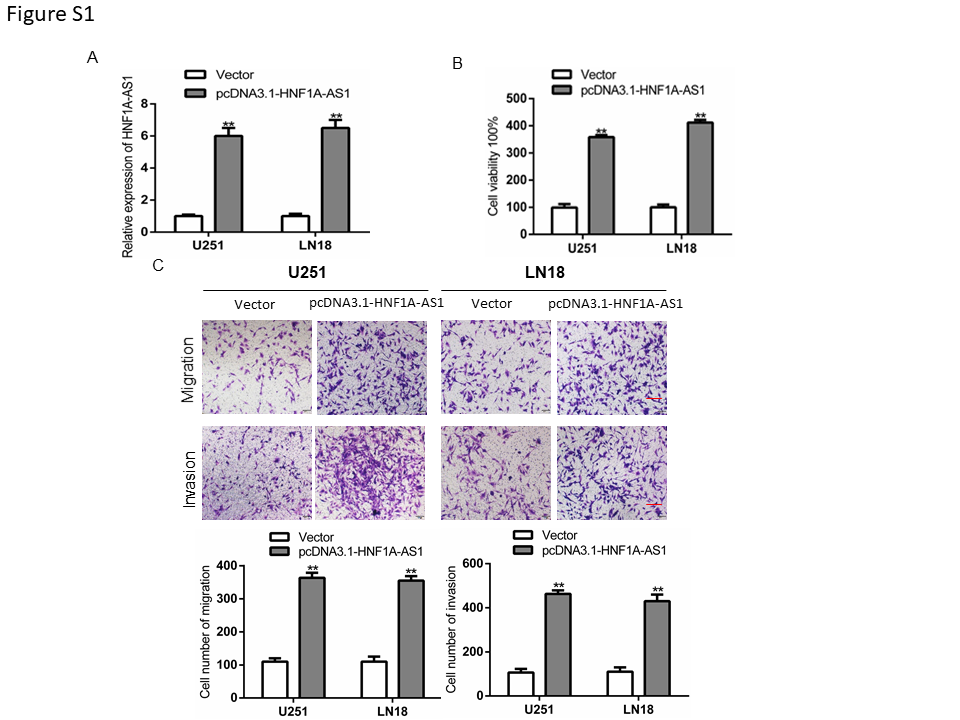

Supplement: Supplementary file 2 — Figure S1 [file 41420_2021_734_MOESM2_ESM.tif]

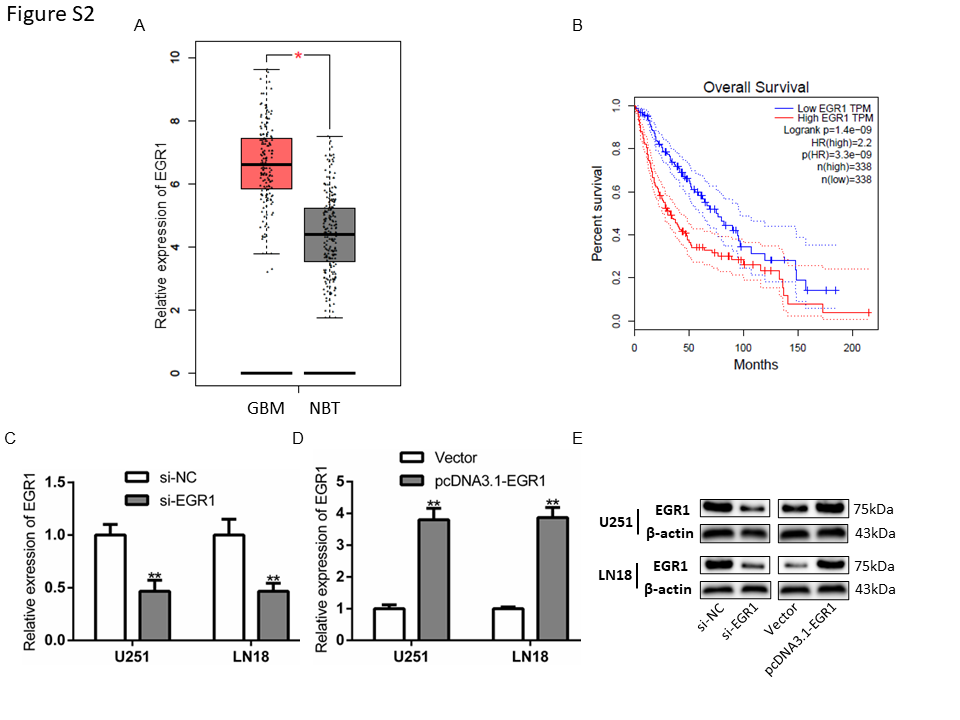

Supplement: Supplementary file 3 — Figure S2 [file 41420_2021_734_MOESM3_ESM.tif]

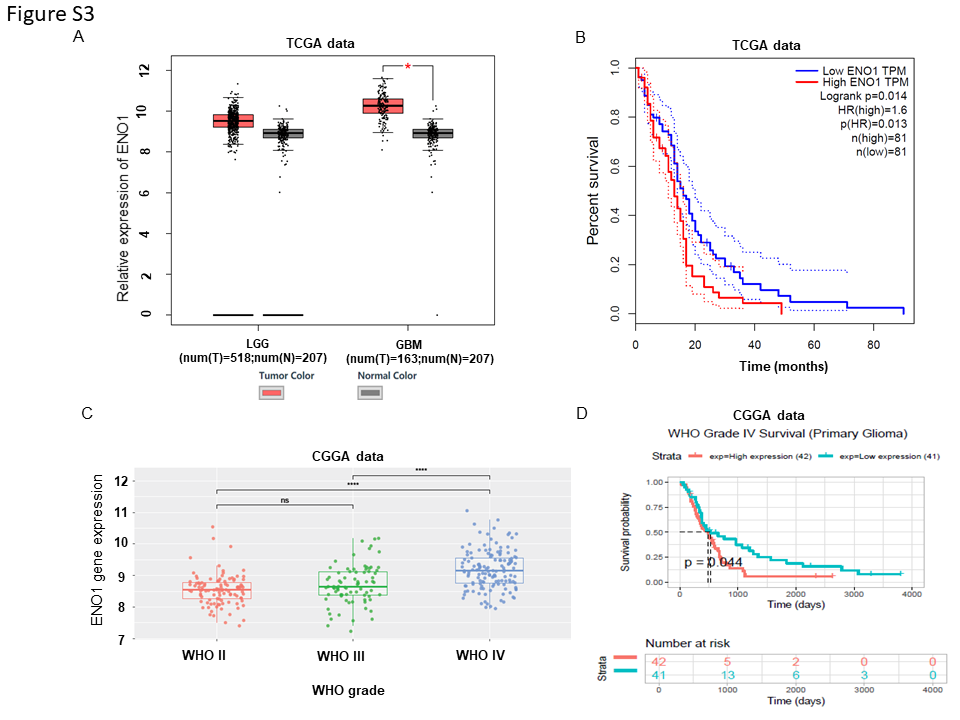

Supplement: Supplementary file 4 — Figure S3 [file 41420_2021_734_MOESM4_ESM.tif]

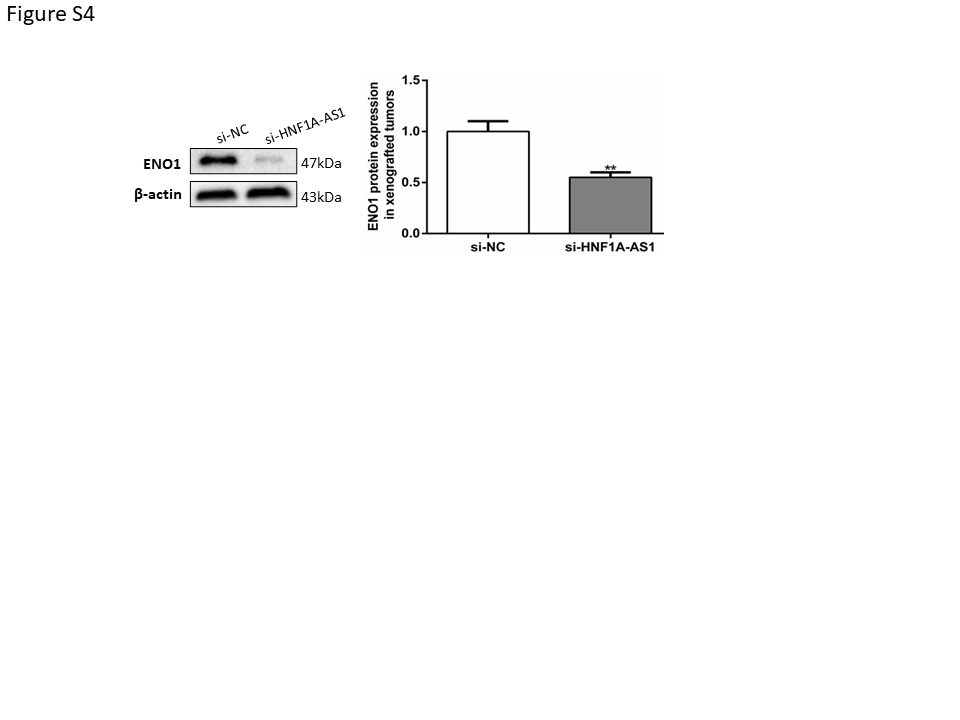

Supplement: Supplementary file 5 — Figure S4 [file 41420_2021_734_MOESM5_ESM.tif]
