# Supplementary material for: EGR1 modulated LncRNA HNF1A-AS1 drives glioblastoma progression via miR-22-3p/ENO1 axis
Source: Cell Death Discov. 2021 Nov 12;7:350. doi: 10.1038/s41420-021-00734-3 (PMC8590016; doi:10.1038/s41420-021-00734-3)
Supplement: Supplementary file 6 — Attribution of Authorship [file 41420_2021_734_MOESM6_ESM.docx]

**Author contributions**

CCM and HLW conceived and designed the experiments. GZ performed the experiments. FY played an important role in interpreting the results. EBB, and JH analyzed the data. YYW drafted the manuscript. ZHY performed the bioinformatics analysis. BZ supervised the whole work and revised the manuscript. All authors read and approved the final manuscript.
